# Associations between the spiritual well-being (EORTC QLQ-SWB32) and quality of life (EORTC QLQ-C30) of patients receiving palliative care for cancer in Cyprus

**DOI:** 10.1186/s12904-021-00830-2

**Published:** 2021-08-30

**Authors:** Maria Kyranou, Marianna Nicolaou

**Affiliations:** 1 Department of Nursing, Faculty of Health Sciences, Cyprus University of Technology, 15 Vragadinou Street, 3603 Limassol, Cyprus; 2 Bank of Cyprus Oncology Centre, 32 Acropoleos Ave., 2006 Strovolos, Nicosia Cyprus

**Keywords:** Cancer, Spiritual well-being, Quality of life, Palliative care

## Abstract

**Background:**

Spiritual well-being is increasingly investigated in relation to patients’ perceived quality of life and is generally thought as having the potential to support patients with cancer who receive palliative care. Until recently, questionnaires used to assess spiritual well-being were developed mainly in the US. The purpose of this study was to translate and use the EORTC- SWB32, a newly developed tool, validated recently in 4 continents, 14 countries, and in 10 languages, to explore relationships of spiritual well-being with quality of life in patients with cancer.

**Methods:**

One hundred four patients participated in this study with an average age of 59 years. Of those, 79% were dealing with metastatic cancer. Data collection took place in three oncology centers from two large cities in Cyprus. The acceptability of the translated items was tested. Two questionnaires were employed for the assessment of quality of life and spiritual well-being, developed by the same organization: the EORTC QLQ-C30 and the EORTC QLQ-SWB32. The scores for each tool were analyzed separately and correlations between the two measures were explored.

**Results:**

Patients found the items of the SWB32 tool easy to understand and answer. They attested that filling the questionnaire prompted thoughts about their own spirituality. The mean score for Global Spiritual Well-Being was 60.4 (SD = 23.7) and it was associated with the mean scores in the scales “Emotional functioning” and “Cognitive functioning” of the EORTC-QOL-C30 (0.42 and 0.40 respectively, *p* < 0.01). The mean score for the “Relationship with God” scale (74.9, SD = 29.7) reported by the Cypriot patients is high and compatible with the homogenous spiritual orientation of the island’s population.

**Conclusions:**

All subscales of the SWB32 tool demonstrated good internal consistency in this study. Significant associations were observed between dimensions of quality of life and spiritual well-being. Additionally, the participants found the items easy to answer consistent with the tool’s suggested clinical utility which lays the ground for the application of targeted interventions to enhance spiritual well-being.

## Background

When this study began, back in 2019, little did we know of what laid ahead of us. The pandemic of COVD-19 commenced in the early months of 2020, and today, after one year of quarantine and social distancing, people around the world are confronted with serious physical and mental health challenges. Despite the advances in the diagnosis and treatment of the disease, health care systems, in most countries, got overwhelmed by the admission rates and health care professionals are facing mass trauma themselves [1]. In 2021, due to the worldwide repercussions of the COVID-19 pandemic, one could potentially question whether patients’ spiritual well-being should still be listed high among nursing research priorities.

Nonetheless, spiritual care is listed in the Code of Ethics of various national and international nursing bodies [1–3]. More recently, both the American Nurses Association incorporates spiritual care in the Scope and Standards of Nursing Practice and the American Association of Colleges of Nursing has integrated spiritual care in the Essentials of Baccalaureate Education. More importantly, people experience greater spiritual needs in times of illness and hospitalization and their spiritual beliefs are perceived by most as a powerful resource to find meaning and hope [2]. Furthermore, research studies attest that attending to the spiritual needs of patients may enhance one’s sense of well-being [3–5] and, as such, it can have a positive impact on quality of life.

Quality of life has been used extensively as an outcome variable in health care studies to reflect patients’ perceptions of well-being as opposed to crude indicators of morbidity and mortality [6, 7]. It has been argued generally that studies which use quality of life as an endpoint should take people’s religious, spiritual and/or existential concerns into account, since such concerns play a role in individuals’ assessments of their quality of life [8]. However, spirituality scholars went a step further and conceptualized spirituality as part of a quality of life framework as proven by multidimensional quality of life instruments (FACIT-Sp.) [9], the QOL-CS Instrument [10], the MPS Scale [11], the WHOQOL-100 [12], the MQOL Scale [13], and the FACT-G [14]. Despite the commonly assumed contribution of spirituality in the multidimensional conceptualization of quality-of-life construct, metanalytical findings argue that spirituality is best seen as a concept that is predictive of quality of life, but which remains distinct from other related concepts such as physical, social and psychological well-being [15].

Spiritual well-being has been one of the many constructs used to assess patients’ spirituality and its association with QOL. The European Organisation for Research and Treatment of Cancer (EORTC) Quality of Life (QOL) Group has recently completed the validation of a standalone measure of spiritual well‐being (SWB) for cancer patients receiving palliative care: the EORTC QLQ‐SWB32 [16]. The working definition on which this tool is based approaches spiritual well-being in terms of four dimensions: Relationships with Others, Relationship with Self, Relationship with Someone or Something Greater, and Existential [17]. This measure followed a structured cross‐cultural development process to address limitations in previous instruments assessing the same construct [17]. As such, it is expected to contribute to a better understanding between spiritual well-being and quality in life in patients with cancer receiving palliative care.

This paper reports on the results of our pilot study that intended to study: i) the adaptability of the Greek version of the EORTC- QLQSW32, ii) the level of spiritual well-being, quality of life, and potential associations between the two, in Cypriot patients with advanced cancer receiving palliative care. The pilot study is part of the larger validation study of the EORTC-SW32 in Greek which has been extended due to restrictions imposed by the pandemic.

## Methods

### Study design and participants

This cross-sectional, observational study investigated the relationship among spiritual well-being and quality of life among patients with advanced cancer who receive palliative care. The validation study took place in Cyprus where the population is approximately 1,200,000 people with the majority of them belonging in the Greek Orthodox Church of Cyprus (94%) [18]. Patients were recruited from 3 different oncology centers in Cyprus, in the two largest cities of the island. A random convenience sample was selected (*n* = 104 patients) who met the following criteria for participation: i) diagnosed with advanced cancer, ii) receiving palliative care, iii) over 18 years, iv) speaking and understanding Greek, v) physically able and willing to participate.

### Procedure

The data for the pilot study were collected between November 2019 and December 2021. Research nurses working in the oncology units delivered information about the study to potential participants. After informed consent was obtained, the second author scheduled time for the administration of the questionnaires. Following demographic data collection, the QLQ-SWB32 was administered to patients and a discussion about potential difficulties in the understanding of the questions took place. A post-administration clinician debrief was included to collect data on the time taken to complete the QLQ-SWB32 questionnaire and whether the questionnaire was self-administered or administered by the clinician. Additionally, data regarding whether patients found any questions confusing or difficult to answer were also collected in the clinician debrief. Research nurses were instructed to mark items that the patient asked for clarification on or indicated their inability to answer. Because the EORTC SWB32 measure has been found to prompt reflection [19], additional time was allowed for an open discussion on issues related to spirituality. Finally, the EORTC QLQ-C30 was completed by participants.

### Measurements

#### Participants’ characteristics

This questionnaire included nine items on sociodemographic and clinical characteristics. These items included age, sex, employment status, educational status, stage of the disease (2 questions) and four questions for clinical treatment followed hitherto.

#### Spiritual well-being

The European Organization for Research and Treatment of Cancer (EORTC) Quality of Life Group (QLG) has recently developed a measure of Spiritual Well-Being for people receiving palliative care for cancer termed QLQ-SWB32 [16]. The validation study for the tool was conducted in 4 continents, 14 countries, in 10 languages and included 451 participants [16, 20]. The final version of the measure consists of 32 questions, 22 of which are grouped in four scoring scales addressing: i) Relationships with others (six items), ii) Relationships with self (five items), iii) Relationship with someone or something greater (five items), and iv) Existential (six items) plus a single-item scoring scale: Relationship with God (RG), for people who indicate that they now believe or have previously believed in God or in someone or something greater than themselves. A four-point scale (Not at all—A little—Quite a bit—Very much) is used for all the items. These scales are each scored separately; summing scales is not appropriate [21].

In addition, there is a global SWB item, with a seven-point response/scoring scale (from 0 = “do not know or cannot answer”, 1 = “very poor” to 7 = “excellent”). Finally, there are eight non-scoring items which can be used to initiate discussion: (i) three items applicable only to people who indicate belief in God or someone or something greater); (ii) five items applicable to all respondents). The measure has been developed to use on its own, is not symptom-focused, and also differs from typical EORTC measures, and many other assessment/ measurement tools, in that it includes some items for their clinical utility, which are not scored, because scores for those items would not be meaningful. They are solely used to prompt relevant discussions with patients recognizing that talking about spirituality might itself be an intervention [20].

The translation of the tool to Greek/Cypriot was based on the translation manual developed by the EORTC Quality of Life Group [22]. Steps included two forward translations of the questionnaire by native Greek/Cypriot speakers, a reconciliation between the two, and two backward translations by native English speakers fluent in Greek. A report describing the process was sent to the EORTC Quality of Life department. After the translation was approved and before the commencement of the validation study, the questionnaire was “pilot-tested” in 10 patients, including extensive debriefing of the patients to check for comprehensibility. The process was completed without problems and no changes were required. After all comments were disclosed to the EORTC, the translation module was deemed acceptable and the Translation department approved an official Greek translation.

#### Quality of life

The EORTC QLQ-C30 (version 3) is a widely used assessment tool, consisting of 30 questions that assess the quality of life of patients with cancer in international clinical trials. It is self-administrated and it includes: i) five functioning scales (physical, PF; role, RF; cognitive, CF; emotional, EF; and social, SF); ii) three symptom scales (fatigue, FA; pain, PA; and nausea and vomiting, NV;) iii) six single items (dyspnea, appetite loss, sleep disturbance, constipation, diarrhea, and financial impact of the disease and treatment, and iv) two items for global health status and quality of life scale (GL). All items employ a 4-point Likert scale, ranging from 1 (not at all) to 4 (very much), except for the two items in the global scale, which use a 7-point scoring scale. The EORTC QLQ-C30 has been translated and validated in Greek cancer patients receiving palliative care treatment [23].

### Data analysis

Data analyses were performed using Statistical Packages of Social Sciences (SPSS) software packages (version 25). Descriptive statistics were used to report demographic and clinical characteristics. Additionally, the following parameters were tested according to specific requirements for each test.

#### Acceptability

The acceptability of the QLQ-SWB32 was assessed in terms of response rate and time needed to complete the questionnaire. Cross-cultural validity was evaluated by examining the number of missing records as well as the number and type of questions that participants found confusing or difficult to answer [24].

#### SWB and QOL assessment

The mean scores from the four scales of the QLQ-SWB32 and Global-SWB were transformed into scores from 0 to 100, with 100 indicating the best possible score for spiritual well-being [20]. For the EORTC QLQ-C30, the mean scores for the scales and single items were linearly transformed to values between 0 and 100 and the mean and standard deviation of each scale/single item were calculated [25]. A higher score for a functioning scale represents a healthier level of functioning, a higher score for the global health status scale represents a higher QOL, and a higher score for a symptom scale/item represents a worse level of symptomatology.

The internal consistency of each scale of the questionnaires was assessed using Cronbach’s α-coefficient, where α-coefficient values ≥ 0.7 indicated adequate scale reliability of the tool [26]. Construct validity was assessed by examining the correlations among subscales of the questionnaires by Pearson’s correlation coefficient (r).

## Results

### Participants characteristics

The characteristics of the patients are listed in Table 1. The mean age of the participants was 58.8 years (SD = 13.7). The sample comprised of slightly more females (57%) than males (43%). Almost half of the participants were retired (47%). Also, 79% of the participants were dealing with metastatic cancer, 48% had more than 4 cycles of chemotherapy and for 39% the tumor was at stage 4. No significant differences were found between age, sex and other characteristics of the participants.

**Table 1:** Differences between study participants’ characteristics and sex^a^

	**Total** ***n* = 104**	**Men** ***n* = 45** **(43%)**	**Women** ***n* = 59** **(57%)**	***p*-value**
**Age**	58.8 (13.7)	58.1 (14.1)	59.4 (13.5)	0.6
**Employment status**				0.3
Unemployed	4 (3.8%)	1 (2.2%)	3 (5.1%)	
Part time	4 (3.8%)	1 (2.2%)	3 (5.1%)	
Full time	22 (21%)	12 (27%)	10 (17%)	
Sick leave	25 (24%)	14 (31%)	11 (19%)	
Retired	49 (47%)	17 (38%)	32 (54%)	
**Educational status**				0.9
High School	45 (47%)	20 (48%)	25 (46%)	
College	19 (20%)	9 (21%)	10 (19%)	
University	32 (33%)	13 (31%)	19 (35%)	
**Stage**				0.1
Stage 1	3 (2.9%)	1 (2.2%)	2 (3.4%)	
Stage 2	23 (22%)	10 (22%)	13 (22%)	
Stage 3	37 (36%)	11 (24%)	26 (44%)	
Stage 4	41 (39%)	23 (51%)	18 (31%)	
**Metastatic cancer**				0.6
YES	22 (79%)	10 (71%)	12 (86%)	
NO	6 (21%)	4 (29%)	2 (14%)	
**Did you have surgery?**				0.5
YES	93 (89%)	39 (87%)	54 (92%)	
NO	11 (11%)	6 (13%)	5 (8.5%)	
**If YES, how many?**				0.4
One	62 (67%)	24 (62%)	38 (70%)	
More than one	31 (33%)	15 (38%)	16 (30%)	
**Chemotherapy cycle:**				0.7
1^st^	20 (19%)	6 (13%)	14 (24%)	
2^nd^	15 (14%)	8 (18%)	7 (12%)	
3^rd^	4 (3.8%)	2 (4.4%)	2 (3.4%)	
4^th^	15 (14%)	6 (13%)	9 (15%)	
More than 4	50 (48%)	23 (51%)	27 (46%)	

^a^Using independent sample t-test for age, and Chi-Square test for categorical variables to compare differences between men and women

### Acceptability

All participants answered all items (response rate 100%, no missing data). Most patients also reported that the questions were clear and easy to understand while 3–7% of patients found at least one question confusing or difficult to answer (mainly items 2, 27, 30, 31). The time needed to complete the questionnaire was 20 min. No emotional reactions were observed while completing the questionnaire. On the contrary, participants expressed appreciation for the opportunity, while filling the questionnaire, to reflect on spiritual matters consistent with the clinical utility of the tool, as suggested by their developers [20].

The majority of patients who answered questions 22 and 23 (“I believe in God or in someone or something bigger than myself” and “I always believed in God or in someone or something bigger than myself”) focused on the meaning of God in the context of their religion, considering spirituality and religiosity as synonyms. Also, it is worth mentioning that all patients answered that they had worries about the future of people who are important to them. Only one patient who answered that he “does not believe in God or feel connected to something greater” and “does not believe in life after death” expressed the opinion that all the questions were unnecessary. He stated: “I prefer to deal with the situation as it is, objectively”.

### Spiritual well-being

Table 2 presents the level of internal consistency for each scale of the SWB32 as assessed with the Cronbach’s α-coefficient. All subscales had satisfactory levels of internal consistency (> 0.7) which indicates adequate scale reliability of the tool. Of the EORTC QLQ-SWB32 subscales, “Relationship with Others” showed the highest mean score (82.3 ± 18.9), followed by “Relationship with God” (74.9 ± 22). The lowest mean score was for “Relationship with Self” (45.2 ± 23.7). The mean score of Global Spiritual Well-Being was 60.4 (SD = 23.7) (Table 2).

**Table 2:** Scores and Cronbach’s alpha-coefficient values for each scale/item in the EORTC QLQ-SWB32

	**Mean**	**SD**	***Cronbach’s alpha***
Relationship with others	82.3	18.9	0.8
Relationship with self	45.2	23.7	0.7
Relationship with someone or something greater	64.6	22	0.7
Existential	69.7	22	0.8
Relationship with god	74.9	29.7	1
Global spiritual well being	60.4	28.7	1

*SD* standard deviation

^a^A high score for the sub-scales indicates better spiritual well-being

Independent Sample t-tests were used as well as Pearson correlation analysis to test sex and age differences in all the subscales of the SWB32 (Tables 3 and 4). There was no significant difference between sex (Table 3) and age (Table 4) in the subscales of the SWB32 (*p* > 0.05).

**Table 3:** Associations between sex and the subscales of the QLQ-C30 and SWB32

	**Total** ***n* = 104**	**Men** ***n* = 45**	**Women** ***n* = 59**	*p*-value*	**Effect size (Cohen’s D)**
***QLQ-C30***					
Global health status/Quality of life^a^	45.2 (24.0)	44.3 (23.8)	45.9 (24.4)	0.7	0.07
Functional scales^a^					
Physical functioning	52.1 (29.5)	61.3 (29.5)	45.1 (27.7)	0.005	-0.6
Role functioning	41.5 (34.3)	43.7 (35.6)	39.8 (33.5)	0.6	-0.1
Emotional functioning	60.3 (29.8)	66.9 (25.2)	55.2 (32.1)	0.04	-0.4
Cognitive functioning	63.1 (30.8)	70.7 (24.7)	57.3 (33.8)	0.02	-0.5
Social functioning	47.4 (34.6)	56.7 (29.8)	40.4 (36.5)	0.01	-0.5
Symptoms scales^b^					
Fatigue	67.2 (30.0)	58.0 (30.4)	74.2 (27.9)	0.007	0.6
Nausea / vomiting	21.5 (32.2)	13.3 (22.6)	27.7 (37.0)	0.02	0.5
Pain	57.7 (36.2)	49.3 (35.9)	64.1 (35.4)	0.04	0.4
Dyspnea	53.8 (39.5)	46.7 (40.5)	59.3 (38.2)	0.1	0.3
Insomnia	57.7 (36.6)	53.3 (35.8)	61.0 (37.2)	0.3	0.2
Loss of appetite	42.3 (38.1)	31.1 (32.1)	50.8 (40.3)	0.006	0.5
Constipation	33.7 (36.1)	37.8 (36.7)	30.5 (35.7)	0.3	-0.2
Diarrhea	24.0 (34.0)	21.5 (31.9)	26.0 (35.6)	0.5	0.1
Financial difficulties	34.0 (36.9)	25.9 (30.1)	40.1 (40.5)	0.04	0.4
***EORTC SWB32***					
Relationship with others	82.3 (18.9)	82.7 (17.8)	81.9 (19.8)	0.8	-0.04
Relationship with self	45.2 (23.7)	50.1 (22.3)	41.5 (24.2)	0.063	-0.4
Relationship with someone or something greater	64.6 (22.0)	61.8 (20.9)	66.8 (22.7)	0.2	0.2
Existential	69.7 (22.0)	73.2 (21.8)	67.0 (21.9)	0.2	-0.3
Relationship with god	74.9 (29.7)	70.7 (30.9)	78.0 (28.6)	0.2	0.3
Global spiritual well being	60.4 (28.7)	62.4(27.8)	58.9 (29.5)	0.6	-0.1

Cohen’s D effect size refers to the magnitude of the difference between sexes, where d ~ 0.2 = low, d ~ 0.5 = medium, και d ~ 0.8 +  = large difference

^a^A high score for the global health status/quality of life and the functional scales represents a high quality of life and a high/healthy level of functioning

^b^A high score for the symptom scales represents a high level of symptomatology/problems

**Table 4:** Pearson correlations of age and the subscales of the QLQ-C30 and SWB32

	Pearson correlations with age
**QQLQ C30**	
Global health status/Quality of life*	-0.13
Functional scales*	
Physical functioning	-0.22*
Role functioning	-0.01
Emotional functioning	-0.09
Cognitive functioning	-0.09
Social functioning	-0.19
Symptoms scales^a^	
Fatigue	0.18
Nausea / vomiting	0.04
Pain	0.04
Dyspnea	0.04
Insomnia	0.04
Appetite loss	0.26**
Constipation	-0.12
Diarrhea	0.16
Financial difficulties	0.07
**SWB 32**	
Relationship with others	-0.09
Relationship with self	-0.09
Relationship with someone or something greater	-0.04
Existential	0.11
Relationship with god	-0.12

^*^p < 0.05, **p < 0.01

^a^A high score for the sub-scales indicates better spiritual well-being

### Quality of life

Table 5 presents the level of internal consistency for each scale of the EORTC-QLQ-C30 as assessed with the Cronbach’s α-coefficient. All subscales had satisfactory levels of internal consistency (> 0.7) which indicates adequate scale reliability of the tool. The participants scored a global health status/QOL scale (GL) mean score of 45.2 (SD = 24). Functional scale scores ranged from 41.5 ± 34.3 for “Role functioning” to 63.1 ± 30.8 for “Cognitive functioning”. Symptom scales ranged from 21.5 ± 32.2 for nausea/vomiting to 67.2 ± 30 for fatigue (Table 5).

**Table 5:** Scores and Cronbach’s alpha-coefficient values for each scale/item in the EORTC QLQ-C30

EORTC-QLQ-C30	Mean	SD	Cronbach’s Alpha
Global health status/Quality of life^a^	45.2	24	0.9
Functional scales^a^			
Physical functioning	52.1	29.5	0.9
Role functioning	41.5	34.3	0.9
Emotional functioning	60.3	29.8	0.9
Cognitive functioning	63.1	30.8	0.7
Social functioning	47.4	34.6	0.8
Symptoms scales^b^			
Fatigue	67.2	30	0.9
Nausea / vomiting	21.5	32.2	0.9
Pain	57.7	36.2	0.9
Dyspnea	53.8	39.5	1
Insomnia	57.7	36.6	1
Loss of appetite	42.3	38.1	1
Constipation	33.7	36.1	1
Diarrhea	24	34	1
Financial difficulties	34	36.9	1

*SD* standard deviation

^a^A high score for the global health status/quality of life and the functional scales represents a high quality of life and a high/healthy level of functioning

^b^A high score for the symptom scales represents a high level of symptomatology/problems

Independent Sample t-tests were used as well as Pearson correlation analysis to test sex and age differences in all the subscales of the QLQ-C30 (Tables 3 and 4). According to the answers to the subscales of QLQ-C30, men reported higher levels of “Physical functioning” than women (*p* = 0.005), better “Emotional functioning” (*p* = 0.04), better “Social functioning” (*p* = 0.01) and better “Cognitive functioning” (*p* = 0.02). Men also experienced less Fatigue (*p* = 0.007), lower levels of Nausea (*p* = 0.02), lower level of “Appetite loss” (*p* = 0.006) and less financial problems (*p* = 0.04) (Table 3). In the QLQ-C30, age had a low negative correlation with “Physical functioning” (*r* = -0.22, *p* = 0.022) and a low positive correlation with “Loss of appetite” (*r* = 0.26, *p* = 0.007) (Table 4).

### Correlations between the SWB32 and QLQ-C30 questionnaires

As shown in Table 6, “Relationship with Self” has a low, positive correlation with “Global Health Status/Quality of Life” (*r* = 0.28, *p* < 0.01), and “Physical functioning” (*r* = 0.30, *p* < 0.01). Also, it has a medium to high positive correlation with “Emotional functioning” (*r* = 0.56, *p* < 0.01), “Cognitive functioning” (*r* = 0.39, *p* < 0.01) and “Social functioning” (*r* = 0.36, *p* < 0.01). “Relationship with Self” has a medium to high negative correlation with Fatigue (*r* = -0.48, *p* < 0.01), Pain (*r* = -0.37, *p* < 0.01), Dyspnea (*r* = -0.41, *p* < 0.01), Insomnia (*r* = -0.35, *p* < 0.01), and “Financial difficulties” (*r* = -0.44, *p* < 0.01).

**Table 6:** Pearson correlations of SWB32 subscales with QLQ-C30 subscales (*N* = 104)

	**Relationship with others**	**Relationship with Self**	**Relationship with someone or something bigger**	**Existential questions**	**Relationship with God**	**Global—SWB**
**Global health status / quality of life**	-0.12	0.28**	0.12	0.27**	0.18	0.15
**Functional scales**						
Physical functioning	-0.21*	0.30**	-0.06	0.24*	-0.10	0.11
Role functioning	-0.29**	0.16	-0.23*	0.00	-0.06	-0.18
Emotional functioning	0.09	0.56**	0.35**	0.50**	0.13	0.42**
Cognitive functioning	-0.04	0.39**	0.25*	0.36**	0.11	0.40**
Social functioning	-0.31**	0.36**	-0.08	0.20*	-0.14	0.06
**Symptoms scales**						
Fatigue	0.28**	-0.48**	0.08	-0.17	0.03	-0.12
Nausea / vomiting	0.14	-0.16	-0.05	-0.06	-0.03	–0.07
Pain	0.21*	-0.37*	0.09	-0.16	-0.07	-0.14
Dyspnea	0.11	-0.41*	-0.04	-0.19	-0.11	-.0.23*
Insomnia	0.24*	-0.35*	0.04	-0.14	-.04	-0.19
Appetite loss	0.20	-0.16	0.01	-0.11	-0.02	0.01
Constipation	0.18	-0.14	0.05	-0.04	0.04	0.07
Diarrhea	0.12	0.00	0.14	0.04	-0.09	0.06
**Financial difficulties**	0.17	-0.44**	-0.13	-0.26**	0.03	-0.32

^*^p < 0.05, **p < 0.01

“Relationship with others” has a low negative correlation with “Physical functioning” (*r* = 0.21, *p* < 0.01), “Role functioning” (*r* = 0.26, *p* < 0.01), and with “Social functioning” (*r* = 0.31, *p* < 0.01). Also, “Relationship with others” has a low positive correlation with Fatigue (*r* = 0.28, *p* < 0.01), “Pain” (*r* = 0.21, *p* < 0.05), and “Insomnia” (*r* = 0.24, *p* < 0.01). “Existential” questions have a low, positive correlation with “Global health status/Quality of life” (*r* = 0.27, *p* < 0.01), “Physical functioning” (*r* = 0.24, *p* < 0.05), and “Social functioning” (*r* = 0.20, *p* < 0.05). Also, a medium to high positive correlation with “Emotional functioning” (*r* = 0.50, *p* < 0.01), “Cognitive functioning” (*r* = 0.36, *p* < 0.01). “Existential” questions have a medium negative correlation with “Financial difficulties” (*r* = -0.26, *p* < 0.01).

“Relationship with someone or something bigger” has a medium positive correlation with “Emotional functioning” (*r* = 0.35, *p* < 0.01) and “Cognitive functioning” (*r* = 0.25, *p* < 0.01). “Relationship with God” does not have any statistically significant correlations with any of the scales of the QLQ-C30. The Global SWB score has a ppositive correlation with “Emotional functioning” (*r* = 0.42, *p* < 0.01), “Cognitive functioning” (*r* = 0.40, *p* < 0.01), and a negative one with Dyspnea (*r* = -0.23, *p* < 0.05).

## Discussion

Given that spirituality is expressed within the wider cultural context of an individual, it would make more sense to compare studies on this topic within similar ethnic or cultural groups. However, in Cyprus, there is no other study that has previously empirically explored patients’ spiritual well-being and its associations with quality of life. Thus, the evaluation of our findings will be explored in relation to those from studies in other countries.

### Spiritual well-being

Two other studies employed the newly developed EORTC-SWB32 tool and reported on levels of spiritual well-being in patients with advanced cancer and its associations with QOL [27, 28]. The perceived Global SWB score was high in all three samples (Table 7). Additionally, the 104 patients from Cyprus scored higher in the “Existential” scale as well as in the “Relationship with Others” and “Relationship with Something Greater” scale compared to that reported by Rhode (2019), from 451 patients (14 countries), and those reported by Chen (2021), from 705 Chinese patients with gynaecological cancer [27, 28]. However, Cypriot patients scored lower compared to patients from the other countries in the “Relationship with Self” scale as well as in the Global SWB score.

**Table 7:** Comparison in the mean scores in the SWB32 scales between studies

Studies	Kyranou 2021	Chen 2021	Rhode 2019
EORTC QOL-C30	**Mean (SD)**	**Mean (SD)**	**Mean (SD)**
Relationship with Others	82.3 (18.9)	70.69 (13)	72.3 (21.8)
Relationship with Self	45.2 (23.7)	75.22 (11)	59.3 (22.7)
Relationship with Someone or Something Greater	64.6 (22.0)	52.2 (11.8)	59.8 (26.7)
Existential	69.7 (22.0)	68,4 (13.3)	61.2 (23.3
Relationship with God	74.9 (29.7)	*	*
Global SWB score	60.4 (28.7)	72.48 (35)	66.5 (25.2)

^*^Not reported by the authors

Similarly, the mean score for the “Relationship with God” scale (74.9, SD = 29.7), reported for the Cypriot population, is high and probably compatible with the fairly homogenous spiritual orientation of the island’s population which is mostly Greek Orthodox [18]. Given the absence of reports from the other two studies on the scale, “Relationship with God”, mean scores on the “Relationship with Someone or Something Greater” scale from the other two studies are used to compare results from all three studies. The reported values in Rhode (2019) and Chen (2021) (between 50 and 60) [27, 28] are considered in the middle range and are lower compared to those reported by our patients (64.6).

The EORTC-SWB32 measure was developed to facilitate measurement of spiritual well-being in multi-cultural environments and it is certainly useful to have different studies reporting on the same measure. Notwithstanding, the findings need to still be translated with caution. It is hard to make meaningful comparisons between the patients of these studies. Spirituality is part of the culture of each population, whichever way defined. As such, it requires a deep understanding of traditions and social connections to interpret findings accurately. Cyprus and China are certainly different. As noted by Chen (2021), even their study from one centre in China might not be representative of different regions of the same country. In the same way there are probably differences among the 14 countries in the study by Rhode (2019) [27, 28].

Finally, it is worth noting that approximately 20% of the patients appear to have not met the eligibility criteria of advanced cancer receiving palliative care (Table 1). Since the EORTC SWB32 was developed in a palliative population, it would be interesting to test whether the module is acceptable in patients with early disease. This finding will be further explored when the validation study will be complete and larger numbers of participants will allow for more meaningful comparisons between groups.

### Quality of life

The EORTC-QLQ-C30 is a valid and extensive tool for the exploration of the various dimensions of the perceived quality of life of patients. Shorter measures have been developed, particularly for the palliative care setting (i.e., the EORTC-QLQ-C15-PAL) [29]. However, we opted for the former because this report is part of a larger study that aims to validate the Greek version of the EORTC-SWB32 which is itself extensive (30 questions). Thus, an extensive measure of quality of life was chosen to test whether any associations between the two may exist. Furthermore, two recent studies, from non-US or northern Europe origin were selected to compare our findings on quality of life with [30, 31]. The aforementioned studies were selected because participants from the Middle East and Africa might share some cultural influences that makes it interesting to compare with our sample from eastern Europe. Most studies on the topic have been performed in the US and Northern Europe [32]. Additionally, the similarities in the demographic and clinical characteristics between the samples of all three studies allowed for further elaboration.

Our participants’ Global health status/QOL scale (GL) mean score was 45.2 (SD = 24). Compared to that reported by Davda (2021) (53, SD = 27) and by Chaar (2018) (65.81, SD = 16.48) the Global health status/QOL scale score in our sample was lower (Table 8) [30, 31]. This is an interesting finding since in the three studies, patients were similar in terms of stage of disease and mean age. Compared to those in the other two studies, the participants in our study had the lowest stated score in the “Physical functioning” scale despite exhibiting a better profile in several symptoms (i.e., fatigue, pain, dyspnea, insomnia and diarrhea) (Table 8). Interestingly, “Role functioning (63.1, SD = 34.3), and “Social functioning” (47.4, SD = 34.6) in our sample was equally low. It would be interesting to explore in future studies the contribution of each scale (i.e., “Role, Social functioning”) in the prediction of the Global health score/QOL.

**Table 8:** Comparison of mean scores in the EORTC QLQ-C30 scales between studies

Studies	**Kyranou 2021 (Cyprus)** ***n* = 104**	**Davda 2021 (Kenya)** ***n* = 100**	**Chaar 2018 (Lebanon)** ***n* = 105**
EORTC QOL-C30	**Mean (SD)**	**Mean (SD)**	**Mean (SD)**
Age	58.8 (13.7)	53.5	56.9 (16.48)
Stage of disease (III & IV)	75%	81%	> 74%
Global health status/Quality of life^a^	45.2 (24)	53 (27)	65.81 (21.16)
Functional scales^a^			
Physical functioning	52.1 (29.5)	63 (28)	76.91 (17.95)
Role functioning	41.5 (34.3)	55 (35)	79.73 (27.35)
Emotional functioning	60.3 (29.8)	68 (28)	68.04 (29.18)
Cognitive functioning	63.1 (30.8)	63 (32)	78.87 (23.01)
Social functioning	47.4 (34.6)	51 (36)	79.04 (26.60)
Symptoms scales^b^			
Fatigue	67.2 (30)	49 (32)	35.17 (24.41)
Nausea / vomiting	21.5 (32.2)	36 (34)	15.12 (23.82)
Pain	57.7 (36.2)	54 (35)	27.84 (26.87)
Dyspnea	53.8 (39.5)	19 (32)	15.12 (25.47)
Insomnia	57.7 (36.6)	35 (38)	41.92 (32.73)
Loss of appetite	42.3 (38.1)	50 (39)	24.06 (33.24)
Constipation	33.7 (36.1)	30 (35)	20.96 (30.55)
Diarrhea	24 (34)	12 (24)	14.78 (25.44)
Financial difficulties	34 (36.9)	79 (31)	26.46 (34)

^a^A high score for the global health status/quality of life and the functional scales represents a high quality of life and a high/healthy level of functioning

^b^A high score for the symptom scales represents a high level of symptomatology/problems

### Correlations between the SWB32 and QLQ-C30 questionnaires

Although the sample size in our study was small, several associations were observed between the scales of the SWB32 and QLQ-C30 (Table 6). The scale “Relationship with Self” demonstrated significant correlations with all items of the QOL-C30. Similarly, the scale “Relationship with Others” demonstrated significant, mostly negative correlations, in ten out of twelve scales of the QOL-C30 (in “Emotional, Cognitive, Social functioning”). “Existential” questions had positive associations with six out the 12 scales of the EORTC-QOL-C30 [“Global health status/Quality of life” (*r* = 0.27, *p* < 0.01), “Physical functioning” (*r* = 0.24, *p* < 0.05), “Social functioning” (*r* = 0.20, *p* < 0.05), “Emotional functioning” (*r* = 0.50, *p* < 0.01), “Cognitive functioning” (*r* = 0.36, *p* < 0.01)], and a negative correlation with “Financial difficulties” (*r* = -0.26, *p* < 0.01).

Given that the items of the “Existential” scale in the SWB32 encompass positive statements (“I have felt able to deal, at peace, my life fulfilling” etc.) positive associations with the above aspects of quality of life are reasonable. Accordingly, they associate negatively with the question “Financial difficulties) (-0.26, *p* < 0.01) (Table 6). Furthermore, in 16 studies that examined the Meaning/Peace factor (which could be thought to correspond broadly to the “Existential” scale of the SWB32) of various spiritual well-being tools, positive associations with overall QOL were reported (ranges from 0.49 to 0.70) and for physical (ranges from 0.25 to 0.28) and mental health (ranges from 0.55 to 0.73) and remained significant after controlling for demographic and clinical variables [32]. Remarkably, the score of the scale “Relationship with someone or something bigger” only had positive correlations with the scales “Emotional functioning” (*r* = 0.35, *p* < 0.01), “Cognitive functioning” (*r* = 0.25, *p* < 0.01) and a negative association with the scale “Role functioning” (*r* = -0.23, *p* < 0.05) whereas “Relationship with God” did not have any statistically significant correlations with any of the scales of the QLQ-C30.

Interestingly, in our study the “Global Health Status/Quality of Life” was not correlated with SWB total score probably due to the small sample size that did not allow for extensive calculations. Whereas, in the study by Chen (2021), with 705 participants, a positive association was observed between the “Global Health Status/Quality of Life” and the SWB total score (0.468, *P* < 0.01). Even in the study by Rhode (2019), where another measure for quality of life was used (the EORTC-QLQ-C15-PAL) [29], a positive association (0.276, *p* < 0.01) was observed between “Global Quality of Life” in the QOL-C15-PAL and SWB global score in the SWB32 [28]. This would be consistent with early studies supporting that the two dimensions are related [8, 10, 33, 34] both at the scale and factor level [32]. The Global SWB score in our study was associated with the scales “Emotional functioning” and “Cognitive functioning” of the EORTC-QOL-C30 (0.42 and 0.40 respectively, *p* < 0.01). Similar associations were observed by Chen (2021) (0.158, 0.339, *p* < 0.01) pointing to the potential contribution of emotional and cognitive aspects of quality of life in the experience of spiritual well-being [27].

### Strengths and limitations

The strength of the current study is its selection of measures for spiritual well-being and quality of life developed by the same organization (EORTC). It is hoped that the comparisons between the two might lead to more meaningful conclusions for the care of patients with cancer receiving palliative care. Additionally, the translation of a validated tool into Greek/Cypriot will give the opportunity for further research in Greek speaking countries where relevant studies are almost absent. This will lead to the exploration of patients’ spiritual well-being and will allow for meaningful comparisons with those reported by patients in other countries/cultures. Given that both Greece and Cyprus are part of the Southern Mediterranean region it might be interesting to see whether patients with cancer differ from patients in Northern Europe in terms of spiritual well-being. Ideally, conclusions drawn from these comparisons could lead to relevant interventions to facilitate patients’ coping with cancer.

The main limitation of the study is its small sample which only depicts trends in the responses and cannot test statistically significant comparisons or perform multivariate analysis between independent and dependent variables. However, this pilot study is part of a larger validation study of the EORTC-SW32 in Greek which has been extended due to restrictions imposed by the pandemic. Hopefully, upon its completion, more comparisons will be applicable between the spiritual well-being and quality of life of patients before and after the pandemic. Also, the cross-sectional design of this study cannot test causal associations between the variables. The validation of the EORTC SWB32 in different cultures will give the opportunity for the design of randomized controlled trials testing the effect of interventions to alleviate spiritual distress and potentially affect patients’ quality of life.

## Conclusions

All subscales of the translated EORTC SWB32 tool demonstrated good internal consistency in this study. Additionally, the participants found the items easy to understand and answer. Furthermore, a medium score was reported in the “Global Spiritual Well-Being” scale (60.4) and a high score in the scales “Relationship with Others” (82.3/100) and “Relationship with God” (74.9/100). Most importantly, the patients of this study viewed the filling of the questionnaire as an opportunity to reflect on their spirituality. This is consistent with the tool’s suggested clinical utility and lays the ground for the application of targeted interventions to enhance spiritual well-being.

In our sample the “Global Health Status/Quality of Life” was not correlated with the SWB total score. This might be attributed to the small sample size and will be tested further when the validation study will be complete. However, the Global SWB score in our study was associated with the scales “Emotional functioning” and “Cognitive functioning” of the EORTC-QOL-C30 pointing to the potential contribution of emotional and cognitive aspects of quality of life in the experience of spiritual well-being [27]. The score of the scale “Relationship with someone or something bigger” had positive correlations with the scales “Emotional functioning”, “Cognitive functioning” and a negative association with the scale “Role functioning” whereas “Relationship with God” did not have any statistically significant correlations with any of the scales of the QLQ-C30.

The construction of new tools to assess spiritual well-being is definitely an arduous process but it bears the potential to bring attention to critical issues of well-being for patients in palliative care treatment. Using them in various countries and cultures adds to their clinical utility. It is well known that dealing with a chronic illness creates many challenges in various aspects of everyday living. However, drawing from spiritual beliefs to maintain hope and a sense of meaning is an adaptive response that seems to associate with better physical health outcomes [35]. Thus, directing practitioners’ as well as patients’ attention to issues related to spiritual well-being might be itself an intervention for the improvement of patients’ quality of life since it is purported by some to make unique contributions in the prediction of quality of life [36]. For all these reasons, spirituality, this unique human experience, becomes a powerful resource for people with life threatening diseases.

## Data Availability

The dataset supporting the conclusions is available from the corresponding author on reasonable request.

## Abbreviations

CF: Cognitive Functioning
EF: Emotional Functioning
EORTC: European Organisation for Research and Treatment of Cancer
FA: Fatigue
GL: Global health status Quality of life scale
MPS: Mental, Physical, and Spiritual Well-Being Scale
MQOL: McGill Quality of Life Questionnaire
NV: Nausea and vomiting
PA: Pain
PF: Physical Functioning
RF: Role Functioning
QoL: Quality of Life
QLQ-C15-PAL: Quality of Life Questionnaire C15 for palliative care
QLQ-C30: Quality of Life Questionnaire—C30
QOL-CS: Quality of Life – Cancer Survivors Instrument
QLQ-SWB32: Quality of Life Questionnaire—Spiritual Well-Being 32
SF: Social Functioning
SWB: Spiritual Well-Being
WHOQOL-100: World Health Organization Quality of Life assessment
